# Impact of Pharmacological Treatments on Rheumatoid Arthritis-Associated Diffuse Interstitial Lung Disease: A Systematic Review and Meta-Analysis

**DOI:** 10.3390/jpm15060239

**Published:** 2025-06-09

**Authors:** Ariam A. Muarif, Rana Algahtani, Lujain H. Alghamdi, Sarah S. Alghamdi, Lama Al Nemer, Reman Alsaqrah, Yazeed Alsulami, Maha Alsharif, Dana Alznbagi, Lena Aljehani, Ziyad Alsaeedi, Sultan Alghamdi, Taif A. Sayel, Basma Al Ghamdi, Ali Al Bshabshe

**Affiliations:** 1 College of Medicine, King Khalid University, Abha 62529, Saudi Arabia; 442803246@kku.edu.sa (A.A.M.); 442803364@kku.edu.sa (R.A.); 441806690@kku.edu.sa (R.A.); 441800925@kku.edu.sa (M.A.); 2 College of Medicine, University of Jeddah, Jeddah 23218, Saudi Arabia; 1912359@uj.edu.sa (L.H.A.); lalgehani.stu@uj.edu.sa (L.A.); 3 Department of Medicine, College of Medicine, Vision Colleges, Riyadh 13226, Saudi Arabia; 202012050@vision.edu.sa; 4 College of Medicine, King Saud bin Abdulaziz University for Health Sciences, Riyadh 11426, Saudi Arabia; alsulami114@ksau-hs.edu.sa; 5 College of Medicine, King Abdulaziz University, Rabigh 25732, Saudi Arabia; 6 College of Medicine, King Abdulaziz University, Jeddah 80200, Saudi Arabia; zhalsaeedi@stu.kau.edu.sa (Z.A.); ssalehalghamdi0003@stu.kau.edu.sa (S.A.); tefalsayel@gmail.com (T.A.S.); 7 Pulmonology Division, College of Medicine, King Khalid University, Abha 62529, Saudi Arabia; baalgamdi@kku.edu.sa; 8 Adult Critical Care Division, College of Medicine, King Khalid University, Abha 62529, Saudi Arabia

**Keywords:** RA-ILD, methotrexate, leflunomide, anti-TNF inhibitors, abatacept, rituximab, JAK kinase inhibitors, antifibrotic inhibitors

## Abstract

**Background**: Interstitial lung disease (ILD) is a prominent complication in the course of rheumatoid arthritis (RA), with a prevalence ranging from 5% to 60% and several phenotypes. The existing knowledge on the impact of different pharmacological interventions in individuals with rheumatoid arthritis-related interstitial lung disease (RA-ILD) is inconclusive, and this variable response to treatment highlights the need for a personalized approach to the management of RA-associated ILD. Therefore, we aimed to evaluate the therapeutic effect and safety of different pharmacological agents, including conventional synthetic DMARDs (Cs DMARDs), biologic DMARDs (bDMARDs), targeted synthetic DMARDs (Ts DMARDs), and antifibrotic agents, in patients with RA-ILD. **Method**: This systematic review and meta-analysis searched for available randomized controlled trials (RCTs) and prospective cohort studies. A search was performed in the PubMed, Google Scholar, and Cochrane Central Register of Controlled Trials (CENTRAL) databases. Eligible studies comprised those involving hospitalized patients diagnosed with RA-ILD, regardless of concomitant medications, who were of adult age (≥18 years); the studies measured the effect of pharmacological interventions, including methotrexate, leflunomide, tumor necrosis factor inhibitors (anti-TNF), abatacept, rituximab, JAK inhibitors, and antifibrotic agents, compared to placebo or other therapies for RA. **Results**: Out of 446 studies from 2002 to 2024, only 16 were included in this systematic review, including 14 prospective cohort studies and 2 placebo-controlled studies. Unfortunately, no RCTs were found that address our research question. The most relevant studies (*n* = 4) were performed in different countries (mainly Spain and the UK), with sample sizes varying from 23 to 381 patients (total: 2199 patients). The current study reveals that non-anti-TNF biologics were associated with a decreased risk of radiologic progression, while advanced therapies improved disease-related outcomes in patients requiring oxygen therapy. Methotrexate and other DMARDs were found to have inconsistent effects on ILD progression and mortality. **Conclusions**: Our review supports the integration of personalized medicine into the management of RA-ILD. By considering patient-specific factors and therapeutic responses, clinicians can better tailor interventions. We confirmed the high methodological quality of the trials, yielding solid evidence for the clinical management of RA-ILD. This review adds to the existing literature by identifying nintedanib as a potential disease-modifying therapy with the potential to slow the progression of lung disease.

## 1. Introduction

Rheumatoid arthritis (RA) is a chronic autoimmune disease that affects about 1% of the population. Its main manifestations are inflammation and damage to the joints. In addition to joints, RA can also affect other systems in the body, including the lungs. Interstitial lung disease (ILD), a complication of RA, accounts for 5% to 60% of its incidence rate [1]. Advanced age, male gender, high-level specific antibodies, previous poor arthritis control, pulmonary function impairment, and HRCT-defined usual interstitial pneumonia (UIP) have all been associated with a worse prognosis in RA-ILD [2]. As RA-ILD is a heterogenous condition, there is a need for the integration of personalized treatment decisions in the complex setting of disease-modifying antirheumatic drugs (DMARDs), which have a profound effect on ILD; notably, these drugs play a key role in the treatment of rheumatoid arthritis but also have consequences for lung health [3]. While some earlier reports reported the possible involvement of methotrexate (MTX) and leflunomide (LEF) in latent lung toxicity, recent evidence has shown that neither MTX nor LEF represents a major added risk factor for the development of ILD associated with RA. However, patients with underlying chronic lung disease or a history of DMARD use are at increased risk of MTX- or LEF-mediated ILD [4]. The use of both in combination in cases of RA-ILD has met some resistance, despite the well-documented benefits of those therapies in other ILDs. Pirfenidone and nintedanib are two antifibrotic agents known to suppress the progression of RA-ILD and the associated decline in lung function. However, additional studies are needed on their use in early phases of the disease and among patients with less lung involvement. Finally, although the clinical efficacy of antifibrotic agents in RA-ILD has not yet been conclusively demonstrated, the emerging evidence supporting the possible benefit of antifibrotics in this context warrants further investigations into their role in clinical practice [3]. However, since the launch of anti-TNF agents for the treatment of RA, many case reports and studies have suggested an association between these therapies and the appearance or worsening of ILD. This association has been seen with all three of the anti-TNF agents that have been approved: infliximab, etanercept, and adalimumab [5]. In the relevant literature, no study has systematically summarized the impacts of different pharmacological interventions on RA-ILD patients. Thus, this meta-analysis was conducted to review the effects of different therapeutic agents on RA-ILD.

## 2. Materials and Methods

### 2.1. Study Design

This systematic review of the literature was limited to randomized controlled trials (RCTs) and prospective cohort studies. The study was registered in the PROSPERO database with ID number CRD42024573485. Due to the type of research, ethical endorsement was not required.

The databases utilized in this study include PubMed, Google Scholar, and the Cochrane Central Register of Controlled Trials (CENTRAL).

### 2.2. Inclusion and Exclusion Criteria

The systematic review was limited to RCTs and prospective cohort studies of adult patients diagnosed with rheumatoid arthritis-associated interstitial lung disease. The included pharmacological interventions for RA-ILD involved treatment with methotrexate (MTX), leflunomide (LEF), anti-TNF agents, abatacept (ABA), rituximab (RTX), JAK inhibitors, and antifibrotic agents. The comparison groups received either a placebo or unspecified alternative treatments for rheumatoid arthritis. The exclusion criteria were as follows: patients with ILD not confirmed to have RA; the exclusion of specific pharmacological therapies; studies with no comparator group; comparators that were not relevant to rheumatoid arthritis treatment; studies that failed to report on the primary or secondary outcomes of interest; and retrospective, case series, or case report studies.

### 2.3. Outcome Measures

The primary outcomes in this review were focused on the incidence, progression, and mortality of interstitial lung disease. Meanwhile, the secondary outcomes included the improvement or stabilization of symptoms associated with interstitial lung disease. The assessment of quality and the risk of bias of the included studies were measured according to the Methodological Index for Non-Randomized Studies (MINORS). This tool assesses the risk of bias in non-randomized studies, including cohort, case–control, and comparative observational studies. MINORS consists of 12 criteria for noncomparative studies and 8 criteria for comparative studies, with a score per study from 0 (criterion not reported) to 2 (criterion adequate). The maximum conceivable scores are 16 for noncomparative studies and 24 for comparative studies. Two authors independently assessed the quality using the MINORS criteria and resolved any disagreements by consensus [6].

## 3. Results

### 3.1. Findings from the Literature

**Figure 1:**
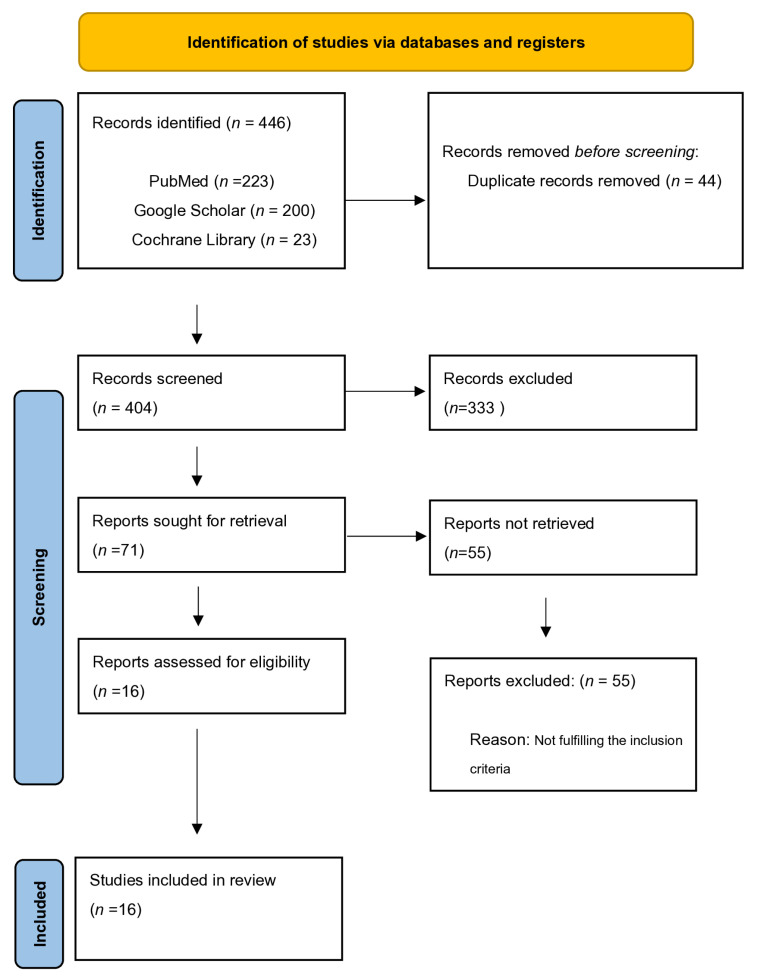
Flow diagram of the studies.

We conducted a systematic search and identified 446 publications that met our inclusion criteria (223 from PubMed database, 200 from Google Scholar, and 23 from Cochrane Library). After the removal of duplicates, 404 articles were retained. A total of 71 full-text articles were assessed for eligibility and included in this systematic review. However, after the application of exclusion criteria, 16 articles published between 2002 and 2024 [1,5,7,8,9,10,11,12,13,14,15,16,17,18,19,20,21] were selected for the final evaluation. The studies included 14 prospective observational cohort studies [1,5,7,8,9,10,11,13,14,15,16,17,18,20,21] and 2 placebo-controlled trials [12,19] (Figure 1).

Unfortunately, none of the included studies were RCTs (see Table 1). The main characteristics of the included articles are shown in Table 1. The total number of participants in the included studies was 2199. The sample size varied between the studies, with a minimum of 23 participants and a maximum of 381 participants. The included articles were conducted in eight different countries, four of which were from Spain [11,13,16,20], and three of which were conducted in the UK [5,14,18] (Table 1). Of these, 14 used a prospective cohort design, which was the most common type. Furthermore, one study was conducted in 15 countries [12]. Only three studies (20%) were conducted in the United States, and the remaining studies were conducted in multiple countries, such as four from Spain (26.7%), two from Italy (13.3%), and one from Switzerland (6.7%). Four studies (26.7%) originated from the UK, whilst two (13.3%) were from the USA. Germany (one study, 6.7%), South Korea (one study, 6.7%), Italy (one study, 6.7%), India (one study, 6.7%), and Greece (one study, 6.7%) were the other countries represented.

**Figure 2:**
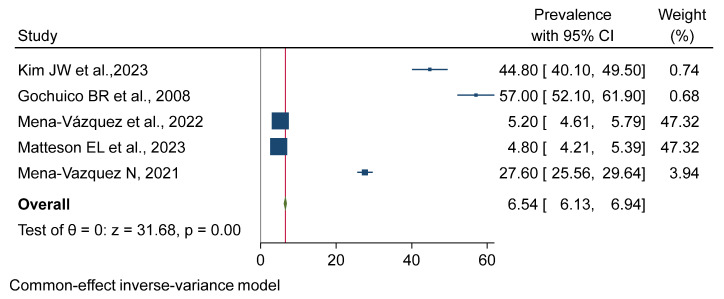
Forest plot for pooled progression rate with treatment among the included studies. (The individual studies display varying prevalence estimates with corresponding 95% confidence intervals, and their weights indicate substantial heterogeneity. The pooled prevalence is 6.54% (95% CI: 6.13–6.94%), supported by a statistically significant z-test (*p* = 0.00). The high weights for Mena-Vázquez et al. and Matteson EL et al., suggest their data heavily influenced the overall estimate) [7,10,11,12,16].

This distribution indicates the worldwide nature of the research contributing to this review. Follow-up over time to assess the outcomes of specific interventions or exposures was reported in three prospective cohort studies [5,7,8]. The high weights for Mena-Vázquez et al., 2022 [11], and Matteson EL et al., 2023 [12], suggest their data heavily influenced the overall estimate.) (see Figure 2).

### 3.2. Patient Characteristics

Most of the studies examined adults (50 to 70 years) [11,20], with a few also including older cohorts. Most participants [1,7,10] had multiple comorbidities, such as chronic obstructive pulmonary disease (COPD), cardiovascular disease, diabetes mellitus, and other neoplasms. A significant percentage of participants also had a history of smoking, as many were current or former smokers, which has previously been linked to the development of respiratory illnesses [14,16]. Different disease durations were examined, and some studies investigated decades-long cases of rheumatoid arthritis (RA) and interstitial lung disease (ILD) [17,18,19]. The follow-up timeframes also varied significantly, ranging from 12 to 60 months. Moreover, differences in sex distribution among cohorts were also noted, with some studies including a higher presence of females compared to studies with a more gender-balanced sample [8,13]. In the risk of bias assessment, the non-randomized studies included in this review were appraised for quality according to the Methodological Index for Non-Randomized Studies (MINORS) [21], with points from 19 up to 23 (maximum 24), yielding an average of 21.125. The study considered at the highest risk of bias was [8] Tardella, 2021, which received a total score of 19. The retrospective cohort study by Druce, 2017 [14], was rated 23, which suggests a much lower risk of bias. In general, the majority of studies presented high methodological quality, with high confidence in interpreting the results (Table 2).

### 3.3. Treatment Received

Rudi T. et al. performed an evaluation of disease-modifying antirheumatic drugs (DMARDs) [1], whereas Kim J. W. et al. [7] reported treatments with methotrexate (MTX, 10.6 mg/week), leflunomide, and tacrolimus. According to Tardella M. et al., abatacept (125 mg/week, subcutaneously) is a bDMARD (biologic DMARD) [8], and Mena-Vázquez et al. [10] studied a treatment duration of ~27 months. Druce K. L. et al. studied rituximab over five years [14]. In the study of Vadillo C. et al., the timeframe studied was 20.6 months [20]. Behera E. K. et al. also studied an antifibrotic agent similar to pirfenidone (1200 mg/day) and nintedanib (150 mg/day) [9], comparing it with a placebo, and Matteson E. L. et al. [12] presented 52-week data with a 17.4-month reporting period in 2022. Detorakis E. E. et al. used anti-TNF-α therapies, infliximab (3 mg/kg), etanercept (50 mg), and adalimumab (40 mg) combined with low-dose MTX (7.5 mg) [17] and other DMARDs for a comparison driven by the research of Dixon W. G. et al. [5], which had a mean follow-up duration of 3.8 years. These include the methotrexate studies of Dawson J. K. et al. [18], a study of a cohort administered doses of 10.7 mg/week for a period of 30 months, and of Kiely P. et al. [15], including a series treated at the same dose of 10 mg for 10 years. Mena-Vázquez N. et al. [13,16] evaluated monotherapy versus a combination therapy approach, inclusive of csDMARDs, bDMARDs, immunosuppressants, and antifibrotics, across a maximum period of 60 months. Comparators were baseline values, placebo, and non-DMARDs; the duration of treatment ranged from 17.4 months to 10 years, and they examined the management of RA-ILD and related conditions. In terms of RA-ILD outcomes and the measurement tools and methods, the majority of studies evaluated clinical decline, stability, or improvement of ILD by conducting serial pulmonary function tests (PFTs), measurements of diffusion capacity for carbon monoxide (DLCO), and high-resolution computed tomography (HRCT). Few studies included clinical exams and biomarkers. However, the timing of these measurements varied widely between studies, with some measuring at baseline and others measuring follow-up periods ranging from every year to every 12–24 months depending on the study design. Cox regression and survival analyses were the statistical methods commonly employed in these analyses to explore the relationship between treatment and ILD outcomes. The treatment efficacy studies were heterogeneous and largely centered around the effects of specific treatments on progression to disease, deaths, stabilizations, and improvements. Rudi T. et al. [1] examined csDMARDs, but did not report the time to progression or mortality rates. Kim J. W. et al. [7] examined three patients being treated with leflunomide (LEF); the time to progression was shorter in patients with ILD treated with LEF than in non-LEF-treated patients. Interestingly, the combination of MTX + tacrolimus was apparently protective in this regard. Tardella M. et al. [8] used high-resolution computed tomography (HRCT), while Zhang et al. [8] examined patients treated with a novel molecule, abatacept (ABA), and noted only a 16% improvement in HRCT compared with 11.4% with HRCT deterioration, with both the stabilization rate and progression rate varying. Behera E. K. et al. [9] examined the impact of antifibrotic medications, with small but statistically insignificant changes in pulmonary function parameters. Through a retrospective analysis, Gochuico B. R. et al. reported that 57% of RA-ILD patients showed improved HRCT results, even though the development of a treatment strategy found an association with worse outcomes [10]. On the other hand, Mena-Vázquez et al. [11] demonstrated stabilization or improvement in 71.9% of patients treated with abatacept, and only 5.2% of patients showed worsening forced vital capacity (FVC) or DLCO. Matteson E. L. et al. found that nintedanib reduced the decline in FVC compared with a placebo, providing the first evidence that nintedanib can attenuate progression in patients with RA-ILD [12]. In addition, Mena-Vázquez N. et al. [13] recorded data from 481 patients with DMARD-treated RA; these data suggested that DMARDs may inhibit ILD progression in many patients in comparison to no treatment and that, for some patients, the disease continued to progress despite DMARD treatment. In terms of mortality, Dixon et al. (2010) [5] reported mortality rates of 68 and 70 deaths per 1000 person-years of follow-up in patients receiving DMARDs and anti-TNF treatments, respectively, but they did not find a direct association between treatment and mortality outcomes. Gochuico et al. [10] reported no mortality data but suggested that prognosis was likely affected by worsening ILD. Mena-Vázquez et al. [13] reported no deaths in their cohort. Moreover, Behera et al. [9,15] and Kim et al. [7] did not include mortality as a primary outcome, instead considering disease progression and the effects of treatment. In terms of safety, the typical side effects of nintedanib and pirfenidone were significantly less pronounced, including gastrointestinal problems such as nausea and vomiting, diarrhea, and decreased appetite. Some patients required hospitalization, especially for respiratory infections, as well as urinary or skin infections. The serious adverse events reported included pneumonia, bronchitis, and liver dysfunction, as well as rare cases of photosensitivity (from pirfenidone) and hypersensitivity pneumonitis. Mena-Vázquez N. et al. reported severe outcomes such as tumor evolution and fast disease progress [16]. Eight of their patients had significant side effects, including three tumors and five deaths due to rapid ILD development.

### 3.4. Meta-Analysis

**Figure 3:**
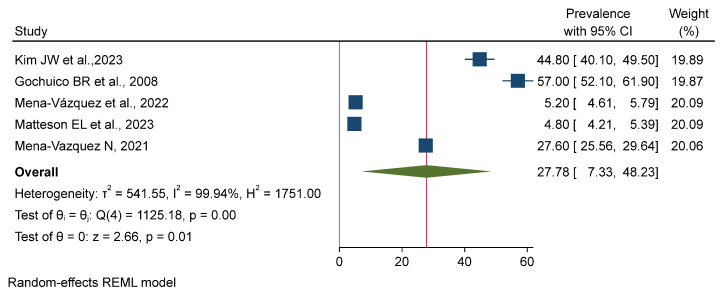
Forest plot for pooled death rate with treatment among the included studies. This forest plot represents a meta-analysis using a random-effects REML model to estimate the pooled prevalence. The prevalence estimates across studies exhibit significant heterogeneity (τ^2^ = 541.55, I^2^ = 99.94%). The pooled prevalence is 27.78% (95% CI: 7.33–48.23%), with a statistically significant overall effect (z = 2.66, *p* = 0.01) [7,10,11,12,16].

**Figure 4:**
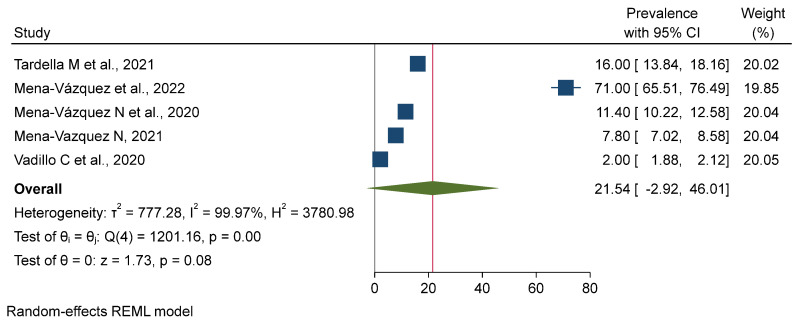
Forest plot for pooled improvement rate with treatment among the included studies [8,11,13,16,20].

There is a very high degree of heterogeneity among the studies, as reflected in the individual prevalence estimates and the corresponding 95% confidence intervals and weights. The overall pooled prevalence was estimated to be 6.54% (95% CI: 6.13–6.94%) as validated by a z-test (*p* = 0.00). The very heavy weights assigned to the studies by Mena-Vázquez et al. [11] and Matteson E. L. et al. [12] indicate that their results have a significant impact on the final estimate. A forest plot was generated from a meta-analysis of the combined prevalence with a random-effects REML model. The analysis found considerable heterogeneity among the studies regarding prevalence (τ^2^ = 541.55, I^2^ = 99.94%). The pooled prevalence was 27.78% (95% CI: 7.33–48.23%), and the overall effect was statistically significant (z = 2.66, *p* = 0.01). The considerable heterogeneity indicates a significant degree of difference between the studies included, highlighting the need for a judicious interpretation of the pooled results (see Figure 2, Figure 3 and Figure 4).

The large body of research that used a prospective cohort design enables the determination of temporal relationships between exposures and outcomes, reducing recall bias. However, residual confounding and selection bias are still profound and major limitations in observational studies. Moreover, the reference to placebo-controlled trials (Matteson E. L. et al. enhances the robustness of the evidence.

## 4. Discussion

We conducted a systematic review of pharmacological treatments for rheumatoid arthritis (RA)-related interstitial lung disease (ILD). Prospective cohort studies represented the most common study design, while only two of the included studies investigated a placebo-controlled trial. The treatments analyzed included conventional disease-modifying antirheumatic drugs (DMARDs), biologic agents such as anti-tumor necrosis factor (anti-TNF) inhibitors, and the newer antifibrotic agents nintedanib and pirfenidone [12]. This review also found that non-anti-TNF biologic agents and antifibrotic agents, especially nintedanib, may help to slow the progression of RAILD and improve lung function. These treatments appear to offer a more tailored approach than regular DMARDs, which have limited efficacy against RA-related pulmonary complications, emphasizing the importance of personalized medicine in the management of such a complex disease. However, direct comparisons between studies are difficult due to differences in the population demographics, outcome measures, and treatment regimens [1]. While a few studies specifically reported improvements in forced vital capacity (FVC) or diffusing capacity for carbon monoxide (DLCO), others assessed mortality or disease progression, producing heterogeneous results. However, even given these differences, the studies show the potential benefit of the early use of biologics or antifibrotic medications to slow RA-ILD progression and improve survival among patients [9]. These results underscore the need for prompt treatment of this potentially serious condition. However, many of the studies included were not randomized controlled trials (RCTs), suggesting the need for further studies with greater rigor. It is critical to conduct RCTs to determine the efficacy of these treatments and identify the best management strategies for RA-ILD. Ongoing studies are planned to address the long-term effects of treatment, comparative clinical trial designs, and the identification of adverse effects in the context of new biologics and antifibrotic agents. All of the studies included were of high methodological quality (risk of bias score: 21.123 ± 1.579 out of 24), indicating the reliability of our findings. One retrospective cohort study was rated using NICE QUADS criteria with a relatively high risk of bias, scoring 19 (of a maximum of 24), thus highlighting a number of limitations within the data [8]. The quality of the studies did not indicate any important biases and supported the findings and conclusions of the reviews, despite the lack of RCTs. This review highlights the encouraging effects of newer pharmacological therapies for RA-ILD, as well as providing a rationale for the design of further translation studies that inform clinical decision making for optimizing the management and clinical outcomes of patients. This review has several strengths. It is the first that incorporates a full meta-analysis of several studies to compute the pooled effects of these treatment strategies on both improvement and mortality in patients with RA-ILD. The random-effects model reduces the impact of the potential heterogeneity of studies in our estimates, leading to more generalizable and robust findings. Furthermore, the inclusion of prospective cohort studies and placebo-controlled trials increases the validity of the findings by blending evidence from observational and interventional studies. However, there are significant caveats to keep in mind. The marked heterogeneity of the pooled mortality rates indicates substantial divergence among the studies included in the analysis, limiting the interpretation of treatment effects. This variability could be due to differences in study populations, treatment protocols, and methods of assessing outcomes. Additionally, while the review discusses the breadth of treatment strategies, the evidence for newer therapies—including JAK inhibitors and antifibrotic modalities—remains scarce. This underscores the necessity of more research to elucidate their roles in the treatment of RA-associated ILD (Figure 4). Finally, the use of corticosteroids in managing patients with an ILD pattern could be a cofounding factor, but this remains uncertain. In a retrospective study conducted by Song et al., half of 84 patients with a UIP pattern who received corticosteroids and immunosuppressive therapy experienced either improvement or stabilization in lung function; however, this was not significantly different from the outcomes observed in the untreated group [22]. Additionally, Lee et al. reported that, among 10 RA-related ILD patients with a UIP pattern, lung function improved in 2 and remained stable in 3 over a median follow-up of 4.2 years [23,24].

## 5. Conclusions

Our review supports the integration of personalized medicine into the management of RA-ILD. By considering patient-specific factors and therapeutic responses, clinicians can better tailor interventions. In the treatment of RA-associated interstitial lung disease (RA-ILD), emerging therapies such as nintedanib have shown promise in slowing the aggressive progression of pulmonary involvement. However, direct comparisons across studies remain challenging due to significant heterogeneity in study designs, patient populations, and outcome assessment methodologies. Continued efforts to harmonize research protocols and broaden the evidence base are essential to optimize treatment strategies focused on more personalized therapeutic approaches and ensure the long-term safety of RA-ILD management.

## Figures and Tables

**Table 1:** Characteristics of the included studies.

Study ID	Title	Study Design	Year of Publication	Country of Origin	Total Number of Patients Included	Number of Patients in Group 1	Number of Patients in Group 2	Outcomes Being Measured
Rudi, 2024 [1]	Impact of DMARD treatment and systemic inflammation on all-cause mortality in patients with rheumatoid arthritis and interstitial Lung Disease: A Cohort Study from the German RABBIT register	Prospective cohort study	2024	Germany	381	DMARD: csDMARD (61), TNFi (114), IL6i (33), T-cell (60) B-cell (64), JAKi (19)	No DMARD (30)	Primary outcome: whether the time to ILD progression is associated with csDMARD exposure. Secondary outcome: whether the time to ILD progression is associated with other clinical variables.
Behera, 202 [9]	Antifibrotics in the Management of Rheumatoid Arthritis-Associated Interstitial Lung Disease: Prospective Real-World Experience From an Interstitial Lung Disease Clinic in India	Prospective observational study	2024	Raipur, India	24	Nintedanib 14	Pirfenidone 10	To study the impacts of antifibrotic drugs on lung function along with standard background therapy in RA-ILD patients.
Matteson, 2023 [12]	Effect of nintedanib in patients with Progressive Pulmonary Fibrosis Associated with Rheumatoid Arthritis: Data from the INBUILD trial	Placebo-controlled trial	2023	N/A	89	Nintedanib 42	Placebo 47	To assess the efficacy and safety of nintedanib versus placebo in patients with progressive RA-ILD in the INBUILD trial.
Kim, 2022 [7]	Methotrexate, Leflunomide And Tacrolimus Use and the Progression Of Rheumatoid-Arthritis-Associated Interstitial Lung Disease	Prospective cohort study	2023	South Korea	143	Patients with ILD progression 64	Patients without ILD progression 79	The primary outcome was the time to ILD progression associated with csDMARD exposure; the secondary outcome was the time to ILD progression associated with other clinical variables.
Mena-Vázquez, 2022 [11]	Safety and Effectiveness of Abatacept in a Prospective Cohort of Patients with Rheumatoid Arthritis–Associated Interstitial Lung Disease	Prospective observational cohort study	2022	Spain	57	At 12 months	End of follow-up	Primary outcome: to evaluate the effectiveness and safety of abatacept for the treatment of RA-ILD in clinical practice. Secondary outcome: to identify risk factors that will help to predict disease progression in patients treated with abatacept.
Matteson, 2022 [19]	Nintedanib in Patients With Autoimmune Disease–Related Progressive Fibrosing Interstitial Lung Diseases: Subgroup Analysis of the INBUILD Trial	Placebo-controlled trial	2022	Rochester, Minnesota,	89	Nintedanib = 42	Placebo = 47	Rate of decline in FVC (ml/year) over 52 weeks; absolute change from baseline in FVC (ml) at week 52; absolute change from baseline in FVC% at week 52.
Tardella, 2021 [8]	Abatacept in rheumatoid Arthritis-Associated Interstitial Lung Disease: Short-Term Outcomes And Predictors Of Progression	Prospective cohort study	2021	Ancona, Italy	44	N/A	N/A	Primary outcome: to evaluate the efficacy and safety of ABA treatment in RA-ILD patients. Secondary outcome: to identify predictors of an unfavorable treatment outcome.
Mena-Vazquez, 2021 [16]	Predictors of Progression and Mortality in Patients with Prevalent Rheumatoid Arthritis and Interstitial Lung Disease: A Prospective Cohort Study	Prospective cohort	2021	Malaga, Spain	116	UIP, n = 71	NSIP, n = 41	(1) Improvement ≥ 10% or DLCO ≥ 15%. (2) Non-progression: FVC ≤ 10% or DLCO < 15%. (3) Progression > 10% or DLCO > 15%. (4) Death.
Vadillo, 2020 [20]	Efficacy of rituximab in Slowing Down Progression of Rheumatoid Arthritis-Related Interstitial Lung Disease: data from the NEREA Registry	Prospective observational cohort study	2020	Madrid, Spain	68	FJD = 22	HCSC = 46	Presence of pulmonary functional impairment.
Mena-Vázquez, 2020 [13]	Non-anti-TNF Biologic Agents Are Associated with Slower Worsening of Interstitial Lung Disease Secondary To Rheumatoid Arthritis	Prospective observational cohort study	2020	Andalucía, Spain	70			To analyze the effects of disease-modifying antirheumatic drugs (DMARDs) on the outcome of interstitial lung disease secondary to rheumatoid arthritis (RA-ILD).
Kiely, 2019 [15]	Is Incident Rheumatoid Arthritis Interstitial Lung Disease Associated With Methotrexate Treatment? Results from a Multivariate Analysis in the ERAS and ERAN Inception Cohorts	Prospective cohort	2019	England, Wales and Ireland	98	MTX-exposed = 39	Non-MTX-exposed = 53	Incidence
Druce, 2017 [14]	Mortality in patients with Interstitial Lung Disease Treated with rituximab or TNFi as a First Biologic	Prospective cohort	2017	Manchester, UK	352	43 RTX	309 TNFi	Death
Detorakis, 2017 [17]	Evolution of Imaging Findings, Laboratory and Functional Parameters in Rheumatoid Arthritis Patients After One Year of treatment with anti-TNF-α agents.	Prospective cohort	2017	Heraklion, Crete, Greece	86	42 study group	44 control group	Effect of TNF-α inhibitors on airways and lung parenchyma compared to nonbiological disease-modifying antirheumatic drugs (nbDMARDs), with regard to efficacy and safety.
Dixon, 2010 [5]	Influence of anti-TNF therapy on mortality in patients with Rheumatoid Arthritis-Associated Interstitial Lung Disease: results from the British Society for Rheumatology Biologics Register.	Prospective observational cohort study	2010	Manchester, UK	367	DMARDs = 68	Anti-TNF = 299	Mortality in DMARDs and anti-TNF.
Gochuico, 2008 [10]	Progressive Preclinical Interstitial Lung Disease in Rheumatoid Arthritis	Prospective	2008	Bethesda, Maryland	64	RA-ILD 31	RAPF 10	To develop a strategy to improve the outcomes of individuals with RA and ILD.
Dawson, 2002 [18]	Investigation of The Chronic Pulmonary Effects of Low-Dose Oral Methotrexate In Patients With Rheumatoid Arthritis: a Prospective Study Incorporating HRCT scanning and Pulmonary Function Tests	Prospective cohort	2002	Merseyside, UK	128	MRX = 55	Non-MTX = 73	Chronic PF caused by low-dose oral methotrexate.

**Table 2:** MINORS risk of bias assessment.

Study ID	Rudi, 2024 [1]	Kim, 2022 [7]	Tardella, 2021 [8]	Behera, 2024 [9]	Gochuico, 2008 [10]	Mena-Vázquez, 2022 [11]	Mena-Vázquez, 2021 [16]	Mena-Vázquez, 2020 [13]	Druce, 2017 [14]	Kiely, 2019 [15]	Detorakis, 2017 [17]	Dixon, 2010 [5]	Dawson, 2002 [18]	Vadillo, 2020 [20]	Matteson, 2023 [12]	Matteson, 2022 [19]
Study Design	Cohort	Cohort	Cohort	Cohort	Cohort	Cohort	Cohort	Cohort	Cohort	Cohort	Cohort	Cohort	Cohort	Cohort	Placebo-controlled trial	Placebo-controlled trial
(1) A clearly stated aim	2	2	2	2	2	2	2	2	2	2	2	2	2	2	2	2
(2) Inclusion of consecutive patients	2	2	2	2	2	2	2	2	2	2	2	2	2	2	1	1
(3) Prospective collection of data	2	2	2	2	2	2	2	2	2	2	2	2	2	2	2	2
(4) Endpoints appropriate to the aim of the study	2	2	2	2	2	2	2	2	2	2	2	2	2	2	2	2
(5) Unbiased assessment of the study endpoint	0	2	2	0	2	2	2	0	2	0	0	0	2	0	2	2
(6) Follow-up period appropriate to the aim of the study	2	2	1	2	2	1	1	1	2	2	2	2	2	2	2	2
(7) Loss to follow-up less than 5%	1	0	2	2	2	2	1	2	1	2	2	1	1	2	1	1
(8) Prospective calculation of the study size	2	2	0	2	0	2	2	2	2	2	0	2	0	2	2	2
(9) An adequate control group (comparative studies only)	2	2	1	2	2	2	2	2	2	2	2	2	2	2	2	2
(10) Contemporary groups (comparative studies only)	2	2	2	2	2	2	2	2	2	2	2	2	2	2	2	2
(11) Baseline equivalence of groups (comparative studies only)	1	1	1	2	2	1	1	2	2	1	2	1	1	2	2	2
(12) Adequate statistical analyses (comparative studies only)	2	2	2	2	2	2	2	2	2	2	2	2	2	2	2	2
Total Score	20	21	19	22	22	22	21	21	23	21	20	20	20	22	22	22

## Data Availability

The original contributions presented in this study are included in the article. Further inquiries can be directed to the corresponding authors.

## Abbreviations

The following abbreviations are used in this manuscript:

ILD	Interstitial lung disease
RA	Rheumatoid arthritis
Anti-TNF	Anti-tumor necrosis factor
DMARDs	Disease-modifying antirheumatic drugs

## References

[1] Rudi T., Zietemann V., Meissner Y., Zink A., Krause A., Lorenz H.M., Kneitz C., Schaefer M., Strangfeld A. (2024). Impact of DMARD treatment and systemic inflammation on all-cause mortality in patients with rheumatoid arthritis and interstitial lung disease: A cohort study from the German RABBIT register. RMD Open.

[2] Fu Q., Wang L., Li L., Li Y., Liu R., Zheng Y. (2019). Risk factors for progression and prognosis of rheumatoid arthritis—Associated interstitial lung disease: Single center study with a large sample of Chinese population. Clin. Rheumatol..

[3] Genovese M.C., Kremer J., Zamani O., Ludivico C., Krogulec M., Xie L., Beattie S.D., Koch A.E., Cardillo T.E., Rooney T.P. (2016). Baricitinib in Patients with Refractory Rheumatoid Arthritis. N. Engl. J. Med..

[4] Atzeni F., Boiardi L., Sallì S., Benucci M., Sarzi-Puttini P. (2013). Lung involvement and drug-induced lung disease in patients with rheumatoid arthritis. Expert Rev. Clin. Immunol..

[5] Dixon W.G., Hyrich K.L., Watson K.D., Lunt M., Symmons D.P.M. (2010). Influence of anti-TNF therapy on mortality in patients with rheumatoid arthritis-associated interstitial lung disease: Results from the British Society for Rheumatology Biologics Register. Ann. Rheum. Dis..

[6] Slim K., Nini E., Forestier D., Kwiatkowski F., Panis Y., Chipponi J. (2003). Methodological index for non-randomized studies (Minors): Development and validation of a new instrument. ANZ J. Surg..

[7] Kim J.W., Chung S.W., Pyo J.Y., Chang S.H., Kim M.U., Park C.H., Lee J.S., Lee J.S., Ha Y.-J., Kang E.H. (2022). Methotrexate, leflunomide and tacrolimus use and the progression of rheumatoid arthritis-associated interstitial lung disease. Rheumatology.

[8] Tardella M., Di Carlo M., Carotti M., Giovagnoni A., Salaffi F. (2021). Abatacept in rheumatoid arthritis-associated interstitial lung disease: Short-term outcomes and predictors of progression. Clin. Rheumatol..

[9] Behera A.K., Kumar V., Sharma P., Ganga R., Meher J., Pati S., Sinha K. (2024). Antifibrotics in the Management of Rheumatoid Arthritis-Associated Interstitial Lung Disease: Prospective Real-World Experience From an Interstitial Lung Disease Clinic in India. Cureus.

[10] Gochuico B.R., Avila N.A., Chow C.K., Novero L.J., Wu H.-P., Ren P., MacDonald S.D., Travis W.D., Stylianou M.P., Rosas I.O. (2008). Progressive Preclinical Interstitial Lung Disease in Rheumatoid Arthritis. Arch. Intern. Med..

[11] Mena-Vázquez N., Rojas-Gimenez M., Fuego-Varela C., García-Studer A., Perez-Gómez N., Romero-Barco C.M., Godoy-Navarrete F.J., Manrique-Arija S., Gandía-Martínez M., Calvo-Gutiérrez J. (2022). Safety and Effectiveness of Abatacept in a Prospective Cohort of Patients with Rheumatoid Arthritis–Associated Interstitial Lung Disease. Biomedicines.

[12] Matteson E.L., Aringer M., Burmester G.R., Mueller H., Moros L., Kolb M. (2023). Effect of nintedanib in patients with progressive pulmonary fibrosis associated with rheumatoid arthritis: Data from the INBUILD trial. Clin. Rheumatol..

[13] Mena-Vázquez N., Godoy-Navarrete F.J., Manrique-Arija S., Aguilar-Hurtado M.C., Romero-Barco C.M., Ureña-Garnica I., Espildora F., Añón-Oñate I., Pérez-Albaladejo L., Gomez-Cano C. (2020). Non-anti-TNF biologic agents are associated with slower worsening of interstitial lung disease secondary to rheumatoid arthritis. Clin. Rheumatol..

[14] Druce K.L., Iqbal K., Watson K.D., Symmons D.P.M., Hyrich K.L., Kelly C. (2017). Mortality in patients with interstitial lung disease treated with rituximab or TNFi as a first biologic. RMD Open.

[15] Kiely P., Busby A.D., Nikiphorou E., Sullivan K., Walsh D.A., Creamer P., Dixey J., Young A. (2019). Is incident rheumatoid arthritis interstitial lung disease associated with methotrexate treatment? Results from a multivariate analysis in the ERAS and ERAN inception cohorts. BMJ Open.

[16] Mena-Vázquez N., Rojas-Gimenez M., Romero-Barco C.M., Manrique-Arija S., Francisco E., Aguilar-Hurtado M.C., Añón-Oñate I., Pérez-Albaladejo L., Ortega-Castro R., Godoy-Navarrete F.J. (2021). Predictors of Progression and Mortality in Patients with Prevalent Rheumatoid Arthritis and Interstitial Lung Disease: A Prospective Cohort Study. J. Clin. Med..

[17] Detorakis E.E., Magkanas E., Lasithiotaki I., Sidiropoulos P., Boumpas D., Gourtsoyiannis N., Antoniou K., Raissaki M. (2017). Evolution of imaging findings, laboratory and functional parameters in rheumatoid arthritis patients after one year of treatment with anti-TNF-α agents. Clin. Exp. Rheumatol..

[18] Dawson J.K., Graham D.R., Desmond J., Fewins H.E., Lynch M.P. (2002). Investigation of the chronic pulmonary effects of low-dose oral methotrexate in patients with rheumatoid arthritis: A prospective study incorporating HRCT scanning and pulmonary function tests. Rheumatology.

[19] Matteson E.L., Kelly C., Distler J.H.W., Hoffmann-Vold A., Seibold J.R., Mittoo S., Dellaripa P.F., Aringer M., Pope J., Distler O. (2022). Nintedanib in Patients with Autoimmune Disease–Related Progressive Fibrosing Interstitial Lung Diseases: Subgroup Analysis of the INBUILD Trial. Arthritis Rheumatol..

[20] Vadillo C., Nieto M.A., Romero-Bueno F., Leon L., Sanchez-Pernaute O., Rodriguez-Nieto M.J., Freites D., Jover J.A., Álvarez-Sala J.L., Abasolo L. (2019). Efficacy of rituximab in slowing down progression of rheumatoid arthritis–related interstitial lung disease: Data from the NEREA Registry. Rheumatology.

[21] Blumhof S., Danila M., Sun D., Valentine V., Luckhardt T., De Andrade J., Bridges S., Duncan S. (2019). Survival of Patients with Acute Exacerbations of Rheumatoid Arthritis Associated Interstitial Lung Disease. Am. J. Respir. Crit. Care Med..

[22] Cassone G., Manfredi A., Vacchi C., Luppi F., Coppi F., Salvarani C., Sebastiani M. (2020). Treatment of Rheumatoid Arthritis-Associated Interstitial Lung Disease: Lights and Shadows. J. Clin. Med..

[23] Song J.W., Lee H.K., Lee C.K., Chae E.J., Jang S.J., Colby T.V., Kim D.S. (2013). Clinical course and outcome of rheumatoid arthritis related usual interstitial pneumonia. Sarcoidosis Vasc. Diffus. Lung Dis..

[24] Lee H.K., Kim D.S., Yoo B., Seo J.B., Rho J.Y., Colby T.V., Kitaichi M. (2005). Histopathologic pattern and clinical features of rheumatoid arthritis-associated interstitial lung disease. Chest.

